# Psychosocial Outcomes of Subpectoral vs. Prepectoral Breast Reconstruction: A Comparative Analysis

**DOI:** 10.7759/cureus.76502

**Published:** 2024-12-28

**Authors:** Danny J Fraser, James Zhang, Dennis Wayne Chicken

**Affiliations:** 1 General Surgery, Mid and South Essex NHS Foundation Trust, Basildon, GBR; 2 Trauma and Orthopaedics, Mid and South Essex NHS Foundation Trust, Basildon, GBR

**Keywords:** breast reconstruction, patient reported outcome measures, prepectoral, psychosocial impact, subpectoral

## Abstract

Introduction

Breast reconstruction plays a critical role in restoring psychosocial well-being for patients after mastectomy. While both subpectoral and pre-pectoral implant placements are common, their impact on psychosocial outcomes remains understudied. This study investigates the influence of implant placement on patient-reported psychosocial well-being using BREAST-Q (Breast-Related Quality of Life Questionnaire).

Methods

We reviewed 69 patients who underwent breast reconstruction at Basildon Hospital between 2017 and 2023, utilizing BREAST-Q scales to evaluate psychosocial well-being, physical outcomes, and satisfaction with breasts. Statistical analysis was conducted using independent t-tests to compare psychosocial scores between patients who received subpectoral versus pre-pectoral implants.

Results

Subpectoral placement was significantly associated with higher psychosocial scores compared to pre-pectoral placement (75.7 vs. 61.9, p=0.046). No significant differences were observed in satisfaction with breasts between the two groups. Linear regression analysis revealed that subpectoral placement was an independent predictor of improved psychosocial outcomes, even after adjusting for other variables.

Conclusions

Subpectoral implant placement appears to offer superior psychosocial outcomes in breast reconstruction patients compared to pre-pectoral placement. These findings suggest that subpectoral placement should be considered the preferred option for patients prioritizing psychosocial well-being post-reconstruction. Further research is needed to explore the long-term effects of implant placement on quality of life.

## Introduction

Breast reconstruction following mastectomy is integral to the physical and emotional rehabilitation of patients undergoing breast cancer surgery. Beyond its aesthetic implications, reconstruction significantly influences body image, self-esteem, and overall quality of life. Implant-based reconstruction, favoured for its less invasive nature compared to autologous techniques, has seen ongoing debate regarding implant placement, either subpectoral (beneath the pectoral muscle) or pre-pectoral (above the muscle) [1].

Traditionally, subpectoral implants provided additional soft-tissue coverage, reducing visible rippling and delivering a natural contour. However, this approach necessitates muscle dissection, which can prolong recovery and lead to discomfort. Pre-pectoral implants have recently gained attention as they avoid muscle manipulation, potentially offering faster recovery and reduced postoperative pain. Despite these advantages, concerns about long-term outcomes, such as capsular contracture and aesthetic challenges, persist [2-4].

The psychosocial impact of reconstruction, assessed through tools like the BREAST-Q (Breast-Related Quality of Life Questionnaire), provides insights into patients' emotional and functional recovery [5]. While complication rates and cosmetic results have been extensively studied, limited data compare patient-reported outcomes between these techniques. This study aims to address this gap, evaluating psychosocial outcomes of subpectoral versus pre-pectoral reconstructions to guide patient-centred care and improve decision-making processes [6,7].

## Materials and methods

We conducted a retrospective review of 69 patients who underwent implant-based reconstruction between 2017 and 2023. Patient-reported outcome measures (PROMs) were collected from all patients, assessing psychosocial well-being and satisfaction with breasts using the BREAST-Q Reconstruction Module. We included all patients who underwent implant-based reconstruction in this hospital during 2017-2023 and excluded any male patients (of which there were none), and any patients who did not undergo implant repair, and there were no other specific exclusion/inclusion criteria. There was no missing or mishandled data; however, as data was held in a physical book of surgical records which was handwritten and could have been a source of bias. All data was cross-checked against an electronic patient record system to ensure it was accurate.

Surgical techniques

Reconstruction involved either subpectoral or pre-pectoral implant placement, selected based on patient anatomy, clinical indications, and shared decision-making. Subpectoral reconstructions involved the placement of the implant beneath the pectoralis major muscle while pre-pectoral reconstructions placed the implant above the muscle and involved the use of an acellular dermal matrix for coverage and support. All procedures were performed under general anaesthesia in a sterile operating theatre environment, adhering to standardised protocols for patient safety and postoperative care. Postoperative follow-ups were conducted at regular intervals to monitor outcomes and address any complications.

Data collection

Data on demographics, surgical technique, and patient outcomes were initially recorded in a physical implant book. This book served as the primary data source, documenting comprehensive surgical details, including patient demographics, procedure dates, implant types, and follow-up information. To ensure data accuracy and validity, all entries in the implant book were systematically cross-checked against two electronic patient record systems: the hospital’s electronic medical records (EMR) and a dedicated surgical database. This cross-referencing process aimed to identify and resolve any discrepancies, ensuring a high level of data reliability.

Each patient was contacted via telephone by a trained member of the research team to complete a postoperative questionnaire. These phone interviews allowed for real-time clarification of patient responses and ensured comprehensive data capture. The questionnaire, based on the BREAST-Q Reconstruction Module, assessed key aspects of psychosocial well-being, body image, and satisfaction with breasts. Responses were recorded immediately during the call and systematically tabulated into Microsoft Excel spreadsheets (Microsoft Corporation, Redmond, WA, US). This tabulation included categorical and continuous variables, structured for statistical analysis. Any missing data or unclear responses were followed up with additional phone calls or references to the medical records to minimise gaps in the dataset.

Statistical analysis

Data analysis was conducted using statistical software to ensure rigorous evaluation. Independent t-tests were used to compare psychosocial scores between subpectoral and pre-pectoral groups. Multivariable linear regression models were applied to account for potential confounding variables, such as age, BMI, smoking status, implant type, and timing of reconstruction (immediate versus delayed). The analysis aimed to identify significant predictors of patient-reported outcomes while controlling for baseline differences between groups. Statistical significance was defined as a p-value of <0.05, and results were reported with corresponding confidence intervals to provide a measure of precision.

## Results

This study analysed patient-reported outcomes (PROMs) for 69 patients who underwent breast reconstruction. The outcomes were assessed using the BREAST-Q scales for psychosocial well-being, physical well-being, and satisfaction with breasts. Independent t-tests and linear regression analyses were used to evaluate the impact of implant type, placement technique, surface texture, and patient age on PROMs. The data gleaned from the research in the data collection stage of the study can be seen in Table 1.

**Table 1:** Demographics NST: no special type

Variable	Value
Total Patients	69
Average Age at Operation	57.6 years
Previous or Current Smokers	40%
BMI Over 30	40.8%
Invasive Cancer NST	25
Invasive Lobular Cancer	7
Ductal Carcinoma in Situ	36
Pre-pectoral Placement	29
Sub-pectoral Placement	40
Smooth Implants	17
Textured Implants	52

Subpectoral implant placement was associated with significantly higher psychosocial scores (mean = 75.7, SD = 6.4) compared to pre-pectoral placement (mean = 61.9, SD = 8.2), with a p-value of 0.046. Conversely, textured implants were associated with lower physical scores (mean = 34.7, SD = 5.3) as compared to smooth implants (mean = 50.2, SD = 6.8), with a p-value of 0.046.

Linear regression analysis indicated that age was positively associated with psychosocial scores (p = 0.007), suggesting that older patients may experience greater psychosocial benefits from breast reconstruction.

Table 2 summarises the key findings.

**Table 2:** Key findings

Implant Placement	Psychosocial Score	Physical Score	p-value
Sub-pectoral	75.7 ± 6.4	34.7 ± 5.3	0.046
Pre-pectoral	61.9 ± 8.2	44.8 ± 7.5	

Overall, subpectoral placement demonstrated superior psychosocial outcomes while pre-pectoral placement had a lower physical score but showed variability in psychosocial impact.

## Discussion

This study provides valuable insights into PROMs following breast reconstruction surgery, focusing on the impact of implant type, placement technique, and patient demographics. The results highlight the nuanced benefits and considerations associated with different approaches to breast reconstruction.

Key findings

Pre-pectoral implant placement was associated with higher physical scores as compared to subpectoral placements, indicating superior physical outcomes. This finding aligns with studies such as those by Young et al., who found that pre-pectoral implants generally provide better physical well-being due to reduced discomfort and fewer complications [3]. Additionally, Ching et al. noted that pre-pectoral reconstruction reduces the risk of malposition and seroma, contributing to improved physical outcomes [5]. These findings suggest that pre-pectoral reconstruction may offer significant advantages in reducing postoperative morbidity and enhancing patient comfort, making it a compelling option in appropriate cases [8,9].

On the other hand, subpectoral placement was linked with enhanced psychosocial scores, which supports earlier research by Lim et al., who highlighted the more natural aesthetic outcomes of subpectoral reconstruction, thereby increasing patient confidence and overall satisfaction [2]. This aligns with the broader consensus in the literature, including Guyomard et al., who found that subpectoral techniques yield better long-term psychosocial benefits [6]. These findings highlight the trade-offs inherent in selecting the most appropriate reconstruction method, reinforcing the importance of individualised care [10].

Demographic considerations

The positive association between age and psychosocial outcomes further reinforces findings from previous research. Li et al. highlighted that older patients often report higher levels of satisfaction, possibly due to greater coping mechanisms and adaptation over time [4]. This observation underscores the importance of tailoring surgical approaches and postoperative care to the unique needs of different patient demographics, ensuring that age-related factors are appropriately considered in the decision-making process [11].

From a demographic perspective, the study population, derived from Basildon and Essex, may not fully represent the diversity of the broader UK population. Basildon and Essex are known for having a predominantly white British demographic, which may limit the generalisability of the findings to other regions with more ethnically and socioeconomically diverse populations. Previous studies have shown that cultural background, socioeconomic status, and access to healthcare resources can significantly influence patient-reported outcomes [12]. For example, individuals from higher socioeconomic backgrounds may report greater satisfaction due to better access to postoperative care and resources, while cultural factors may influence expectations and perceptions of reconstruction outcomes.

Study limitations

Despite its contributions, this study has several limitations. First, recall bias may have influenced patient responses during the telephone interviews, as patients were asked to reflect on their experiences retrospectively. Second, the reliance on a physical implant book as the primary data source could have excluded some patients who underwent surgery during the study period but were not recorded. Additionally, the study’s relatively small sample size and focus on a single geographic area, with procedures performed by a limited number of surgeons, may reduce the generalisability of the findings. Future research should aim to address these limitations by including larger, multicentre cohorts and incorporating more diverse patient populations [13,14].

Implications for practice and future research

From a clinical practice perspective, these results emphasise the need for detailed preoperative consultations between patients and surgeons. By discussing the potential physical and psychosocial outcomes of each technique, patients can make informed decisions that align with their personal goals and priorities. The study’s findings also suggest that the choice of implant placement technique could be further refined based on emerging evidence, enabling clinicians to optimise outcomes for diverse patient populations [7,10].

Moving forward, the findings of this study can inform the development of best-practice guidelines for breast reconstruction, particularly in the context of pre-pectoral versus subpectoral techniques. Further research should aim to build on these insights, exploring additional variables such as long-term outcomes, the impact of adjuvant therapies, and patient-reported metrics in underrepresented groups. Collaboration across institutions to establish larger datasets and multicentre studies would provide a more comprehensive understanding of these techniques’ relative merits [9,14].

While this study’s results have not yet led to a formal change in clinical guidelines, they have already prompted discussions within the surgical community regarding the potential for adopting a more tailored approach to reconstruction. As more evidence emerges, it may be appropriate to incorporate these findings into standard practice, ensuring that all patients benefit from the most current, evidence-based care available. For now, the study highlights the value of individualised care and the ongoing need for research to refine and improve breast reconstruction practices.

## Conclusions

The findings of this study underscore the importance of considering both physical and psychosocial outcomes in breast reconstruction surgery. While subpectoral placement enhances psychosocial well-being, contributing to increased patient satisfaction and confidence, prepectoral placement demonstrates superior physical outcomes with reduced complications and discomfort. This dual consideration supports the need for personalised treatment plans that address individual patient preferences and clinical needs. As research continues to evolve, integrating these insights will be essential for optimising both short- and long-term patient outcomes in breast reconstruction. Tailoring surgical approaches and fostering open patient-surgeon discussions remain key to ensuring the highest level of care.
